# Use of Cangrelor in Türkiye: A Multicenter Real-Life Study

**DOI:** 10.3390/jcm14248728

**Published:** 2025-12-10

**Authors:** Servet Altay, İlker Gül, Fatih Aytemiz, Metehan Kibar, Cuma Süleymaoğlu, Halil Fedai, Alp Burak Çatakoğlu, Şükrü Çetin, Selin Yöndem, Mehmet Vefik Yazıcıoğlu, Diyar Köprülü, Mustafa Çetin, Süleyman Sezai Yildiz, Beytullah Çakal, Çağrı Yayla, Selçuk Korkmaz

**Affiliations:** 1 Department of Cardiology, Trakya University Faculty of Medicine, 22030 Edirne, Türkiye; 2 Department of Cardiology, Bakırcay University Cigli Training and Research Hospital, 35620 İzmir, Türkiye; 3 Department of Cardiology, City Hospital of Manisa, 45030 Manisa, Türkiye; 4 Department of Cardiology, Koşuyolu Kartal Heart Training & Research Hospital, 34846 İstanbul, Türkiye; 5 Department of Cardiology, Osmaniye State Hospital, 80000 Osmaniye, Türkiye; 6 Department of Cardiology, Faculty of Medicine, Harran University, 63290 Sanliurfa, Türkiye; 7 Department of Cardiology, Liv Hospital Ulus, 34340 İstanbul, Türkiye; 8 Department of Cardiology, Sehit Prof. Dr. Ilhan Varank Research and Education Hospital, 34785 İstanbul, Türkiye; 9 Department of Cardiology, University of Health Science, Ankara City Hospital, 06800 Ankara, Türkiye; 10 Department of Cardiology, Liv Hospital Vadi Istanbul, 34396 İstanbul, Türkiye; 11 Department of Cardiology, Ordu State Hospital, 52200 Ordu, Türkiye; 12 Department of Cardiology, Mehmet Akif İnan Training and Research Hospital, 63040 Sanliurfa, Türkiye; 13 Department of Cardiology, University of Health Science, Prof. Dr. Cemil Taşçıoğlu City Hospital, 34384 İstanbul, Türkiye; sezai04@yahoo.com; 14 Department of Cardiology, Istanbul Medipol University, 34214 İstanbul, Türkiye; 15 Department of Biostatistics and Medical Informatics, Trakya University Faculty of Medicine, 22030 Edirne, Türkiye; selcukorkmaz@gmail.com

**Keywords:** Cangrelor, PCI, acute coronary syndrome, chronic coronary syndrome

## Abstract

**Background/Objectives**: The only intravenous P2Y12 inhibitor used in the management of acute and chronic coronary syndromes is cangrelor. Previous studies have demonstrated the efficacy of cangrelor. However, limited real-world data are available for cangrelor usage. This study aimed to investigate the use of cangrelor in large-volume percutaneous coronary intervention (PCI) centers in Türkiye, and examine the indications, patient characteristics, bleeding, and ischemic events. The efficacy and safety of cangrelor in this high-risk group were evaluated. **Methods**: This study was conducted retrospectively in 14 high-volume centers in Türkiye with extensive cangrelor experience. Cangrelor indications, patient clinical characteristics, periprocedural and postprocedural treatments, in-hospital and follow-up bleeding, ischemic events, and mortality were analyzed. **Results**: This study recruited 411 patients (mean age: 63.8 ± 12.7 years; 76% male). The most common conditions in which cangrelor is used in Türkiye are cardiogenic shock, intubation and nausea/vomiting, where P2Y12 cannot be used adequately due to impaired oral intake. The incidence rate of any bleeding within 48 h was 6.4% (n = 26), with major bleeding accounting for 1.7% of all cases (n = 7). The bleeding rates were similar between patients aged <75 years and those aged ≥75 years (6.0% vs. 8.8%, *p* = 0.326), as well as between patients with chronic kidney disease (CKD) and those without CKD (6.3% vs. 7.9%, *p* = 0.600). **Conclusions**: This is the first multicenter, large-cohort study to examine cangrelor use in Türkiye, providing real-world evidence for the efficacy and safety in high-risk patients with complex clinical features and lesion characteristics.

## 1. Introduction

Acute coronary syndrome (ACS) is a leading cause of mortality and disability worldwide [1]. According to the current guideline recommendations, early percutaneous coronary intervention (PCI) and stent implantation can aid in mitigating mortality and heart failure rates [2,3]. However, the antiplatelet activities of aspirin and P2Y12 inhibitors must be adequate before PCI to prevent early stent thrombosis, reinfarction, and mortality. The efficacy of commonly used P2Y12 inhibitors (clopidogrel, ticagrelor, and prasugrel) is delayed due to gastrointestinal absorption. Additionally, the effectiveness of these inhibitors may be suboptimal under certain conditions [4,5]. Hypotension, nausea/vomiting, intubation, delayed gastric passage, impaired intestinal perfusion, hypothermia, and opioid use, which are frequently observed in patients with ACS, can limit the efficacy and use of oral P2Y12 inhibitors [6,7,8]. Additionally, 10–15% of patients with ACS may require urgent coronary artery bypass grafting (CABG) post-angiography. The prolonged time required for the activity of oral P2Y12 inhibitors can increase bleeding risk in these patients [5].

The efficacy of cangrelor, an intravenous P2Y12 inhibitor with rapid onset and offset of action [8], has been compared with that of clopidogrel in patients with ACS and chronic coronary syndrome (CCS) undergoing PCI [9,10,11]. Current guidelines recommend cangrelor use with a class 2b indication in patients with ACS who have not received oral P2Y12 inhibitors [2,12]. In Türkiye, cangrelor use in adult patients undergoing PCI who have not received oral P2Y12 inhibitors and in whom oral P2Y12 therapy is not feasible or desirable is prescribed based on the Ministry of Health regulations, which have limited its use to patients with cardiogenic shock or cardiac arrest who are unconscious or intubated or cannot be fitted with a nasogastric tube [13]. This patient group indicates high-risk patients.

This study aimed to evaluate cangrelor use, indications, patient characteristics, bleeding, and ischemic events in large-volume PCI centers in Türkiye.

## 2. Methods

Data for this study were collected between 20 January 2025 and 1 April 2025, at 14 high-volume PCI centers in Türkiye with established 24/7 cangrelor programs. This study is a retrospective study and the data include past five years usage data of cangrelor. All patients who used cangrelor in the centers participating in the study were included in the study.

In this study, 411 patients diagnosed with ACS or CCS who received cangrelor according to approved indications before PCI were included. Patients with non-obstructive coronary arteries, such as myocardial infarction with non-obstructive coronary arteries or Takotsubo syndrome, were excluded [14,15,16]. Data on cangrelor indications, baseline and clinical characteristics, periprocedural details, in-hospital outcomes, and follow-up bleeding, ischemic events, and mortality were collected. Based on the European Society of Cardiology guidelines, patients were categorized into the following groups: ST-elevation myocardial infarction (STEMI), non-ST-elevation myocardial infarction (NSTEMI), unstable angina (UA), and CCS groups [2,3]. Stent thrombosis was defined according to Academic Research Consortium (ARC) criteria as definite or probable events confirmed angiographically or clinically [17].

Bleeding events were assessed at 48 h and 1 month and classified using the Bleeding Academic Research Consortium (BARC) and Global Use of Strategies to Open Occluded Coronary Arteries (GUSTO) criteria.

BARC Classification: Type 1: Bleeding not requiring unplanned tests, hospitalization, or treatment; Type 2: Bleeding requiring non-surgical medical intervention, hospitalization, or increased care; Type 3a: Overt bleeding with 3–5 g/dL hemoglobin drop or transfusion requirement; Type 3b: Overt bleeding with ≥5 g/dL hemoglobin drop, cardiac tamponade, or requiring surgical intervention (excluding dental, nasal, skin, or hemorrhoidal bleeding) or intravenous vasoactive agents; Type 3c: Intracranial or intraspinal bleeding or vision-impairing intraocular bleeding; Type 4: CABG-related bleeding, including perioperative intracranial bleeding, reoperation after sternotomy closure, or significant transfusion requirements; Type 5a: Probable fatal bleeding without autopsy/imaging confirmation; Type 5b: Definite fatal bleeding confirmed using autopsy/imaging. Bleeding events with types 1–2 and types ≥ 3 were considered minor and major events, respectively [18].

GUSTO Classification: Severe/life-threatening: Intracerebral hemorrhage or bleeding causing hemodynamic compromise requiring treatment; Moderate: Requiring transfusion but not causing hemodynamic compromise; Mild: Bleeding not meeting the above criteria [19].

Chronic kidney disease (CKD) was defined as a glomerular filtration rate of <60 mL/min (calculated using the CKD-EPI formula) [20]. Contrast-associated acute kidney injury (CA-AKI) was defined according to KDIGO criteria as an increase in serum creatinine ≥ 0.3 mg/dL within 48 h or ≥1.5 × baseline within 7 days after contrast exposure [21]. Patients receiving chronic dialysis were excluded from CA-AKI analyses. All numeric variables were verified for consistency across centers before analysis, and data audits were performed to ensure accuracy of procedural and outcome variables. In-hospital data were obtained from patient records, while long-term follow-up data were collected from hospital records, telephone interviews, and the national healthcare system (e-nabız) [22]. For survival analysis, the time-to-event variable was defined as the interval (in months) from the index PCI to death or censoring. Death times were obtained from follow-up records; in-hospital deaths without a recorded follow-up duration were assigned survival times equal to the length of hospitalization expressed in months. Patients alive at last contact were censored at their follow-up time, truncated at 12 months. Kaplan–Meier survival curves were generated for each CAD category, and intergroup differences were evaluated using a center-stratified log-rank test. This study complied with the guidelines of the Declaration of Helsinki and was approved by the Trakya University School of Medicine ethics committee (TUTF-GOBAEK-2024/560).

## 3. Statistical Analysis

Continuous variables were summarized as mean ± standard deviation (SD) or median [interquartile range, IQR], depending on distribution. Categorical variables were reported as frequency and percentage. Incidence rates were calculated with 95% confidence intervals using the Wilson method. Bleeding rates according to BARC and GUSTO classifications were summarized by coronary artery disease type (STEMI, NSTEMI, UA, CCS). Time windows (48 h and a 30-day window with ±2-day allowance) were defined relative to the first occurrence of an event. Kaplan–Meier curves were used to estimate 12-month major adverse cardiac event (MACE)-free survival and overall survival, and groups were compared using the log-rank test; stratified log-rank tests were applied when center imbalance was suspected. Event-free patients at follow-up were right-censored. Missingness was evaluated for each variable; predictors included in the multivariable logistic regression model (age, hypertension, diabetes mellitus, CKD, Killip class, P2Y12 inhibitor type, and gender) had <1% missingness, and variables with >30% missingness were excluded. Multivariable logistic regression was used to identify independent predictors of 48 h bleeding, and results were expressed as odds ratios (OR) with 95% confidence intervals. A two-sided *p* < 0.05 was considered statistically significant. All analyses were performed using R version 4.5.1 (R Foundation for Statistical Computing, Vienna, Austria).

## 4. Results

Of the 411 patients included in this study, the median age of patients in the STEMI, NSTEMI, UA, and CCS groups was 64, 70, 66, and 59 years, respectively. Patients in the STEMI group exhibited advanced Killip classes, increased heart rates, and decreased systolic and diastolic blood pressures at presentation. Hypertension prevalence was high in all groups. Patients in the STEMI and NSTEMI groups exhibited increased prevalence of CKD and diabetes, respectively. The lowest median ejection fraction was observed in the STEMI group (Table 1).

Cangrelor use was most common in cases of cardiogenic shock, intubation, and nausea/vomiting due to oral intake impairment. The most common major lesion was located in the left anterior descending artery. In the study cohort, 59% of patients had two or more coronary lesions. The femoral approach was used in 72% of cases, while 95% of patients received drug-eluting stents, predominantly everolimus-eluting stents. All patients received aspirin and heparin. Clopidogrel was the most commonly used P2Y12 inhibitor (57%). P2Y12 was started immediately after the end of cangrelor in all cases. Median stent diameter was 3.0 mm, total stent length was 34.3 ± 19.7 mm, median procedure duration was 39 min (IQR 26–55), contrast volume was 150 mL (IQR 110–220), and cangrelor infusion duration was 2 h (IQR 2–2) (Table 2).

In-hospital events included inotrope requirement (40%), in-hospital mortality (31%), contrast-induced nephropathy (14%), and transfusion requirement (3.9%). The incidences of inotrope use and mortality were significantly high in the STEMI group. Other in-hospital events were similar between the groups.

The incidence rate of any bleeding events within 48 h was 6.4% (n = 26). The major bleeding rate was 1.7% (n = 7), which was not significantly different between the groups. The most common bleeding sites were vascular access and the urinary system. Most major bleeds were located in the gastrointestinal system. Among patients with gastrointestinal bleeding, 57% (n = 4) were being treated with glycoprotein IIb/IIIa inhibitors. Allergic reactions or adverse events requiring cangrelor discontinuation were not noted (Table 3 and Table 4).

The number of bleeding events within 48 h in the ticagrelor/prasugrel-treated group was non-significantly higher than that in the clopidogrel-treated group (*p* = 0.227 for BARC, *p* = 0.307 for GUSTO). After age stratification, the bleeding rates in patients treated with BARC aged <75 years and ≥75 years were 6.0% and 8.8%, respectively. Meanwhile, the bleeding rates in patients treated with GUSTO aged <75 years and ≥75 years were 5.6% and 8.8%, respectively (relative risk (RR): 0.97; *p* = 0.326). Among patients treated with BARC, the bleeding rates in the CKD and non-CKD groups were 6.3% and 7.9% (CKD), respectively. Meanwhile, the bleeding rates in the non-CKD and CKD groups were 6.0% and 7.9%, respectively, among patients treated with GUSTO (RR = 0.98, *p* = 0.600).

**Figure 1:**
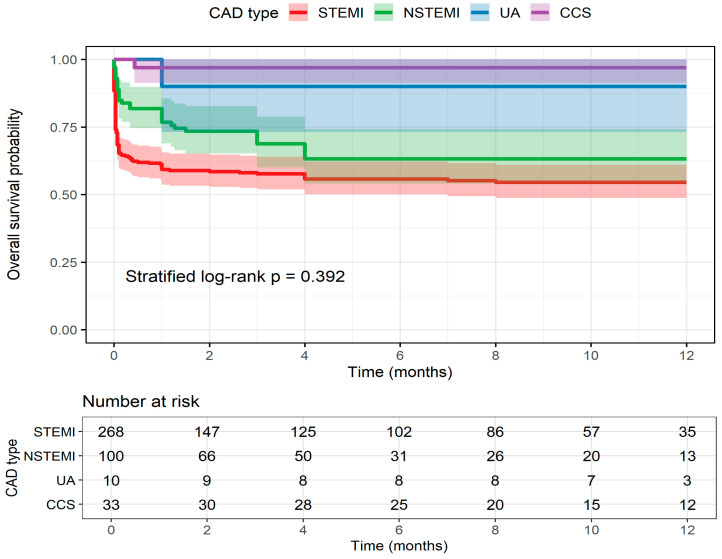
Kaplan–Meier curves showing 12-month overall survival stratified by coronary artery disease (CAD) subtype: ST-elevation myocardial infarction (STEMI), non-ST-elevation myocardial infarction (NSTEMI), unstable angina (UA), and chronic coronary syndrome (CCS). Shaded areas represent 95% confidence intervals. The table below displays the number of patients at risk over time. Differences between groups were assessed using a center-stratified log-rank test.

No significant differences in 12-month overall survival were observed between CAD groups (STEMI, NSTEMI, UA, and CCS; log-rank *p* = 0.392, stratified by center) (Figure 1). At 12 months, Kaplan–Meier estimated survival was 54.6% (95% CI 48.8–61.1) for STEMI, 63.3% (95% CI 54.0–74.1) for NSTEMI, 90.0% (95% CI 73.2–100) for UA, and 97.0% (95% CI 91.3–100) for CCS, with 35, 13, 3, and 12 patients remaining at risk, respectively. Advanced age (≥75 years) and CKD were not associated with bleeding risk within 48 h, despite slightly increased rates in these subgroups.

Age was an independent and significant predictor of 48 h bleeding (odds ratio [OR]: 1.04; 95% CI: 1.01–1.08; *p* = 0.018), indicating that bleeding risk increases with age. The bleeding risk in patients treated with potent P2Y12 inhibitors (ticagrelor/prasugrel) was higher than that in patients treated with clopidogrel (OR: 2.17; *p* = 0.064). However, the wide CI and borderline *p*-value suggest the need for confirmation in larger samples. Hypertension was associated with decreased bleeding risk (OR: 0.32; *p* = 0.007). Cardiogenic shock (Killip 4), diabetes, CKD, and female sex were not significantly associated with 48 h bleeding (Table 5).

The incidence of major ischemic events (myocardial infarction, revascularization, and stent thrombosis) within 48 h and 1 month was 4.2%. Definite stent thrombosis occurred in 9 patients (2.2%) within the first month after PCI, with no additional cases through 12 months. Although ischemic event rates were lower in the ticagrelor/prasugrel group than in the clopidogrel group (RR = 0.75 and 0.76, *p* = 0.770), the difference did not reach statistical significance.

## 5. Discussion

Cangrelor, which is the only intravenous P2Y12 inhibitor, is critical for ACS management [23,24]. The CHAMPION trials established the efficacy of cangrelor. However, real-world data on the efficacy of cangrelor are limited. In Türkiye, cangrelor use is restricted to high-risk patients due to reimbursement regulations. The most common conditions for cangrelor are cardiogenic shock and intubation due to impaired oral intake. The high prevalence of inotrope use and in-hospital mortality in the study cohort indicates that cangrelor is used in very high-risk patients in Türkiye. This multicenter, real-world study provides valuable insights into cangrelor use in high-risk populations, supporting recent discussions on its role in managing cardiogenic shock [25,26,27].

The mean age, male predominance, and smoking prevalence of the study cohort are consistent with the characteristics of previous ACS studies in Türkiye. However, this study reported increased incidence rates of hypertension and diabetes when compared with previous studies [28,29]. Compared with international studies, the study cohort exhibited a high burden of risk factors, especially smoking and diabetes [30,31,32].

Cangrelor is most frequently preferred worldwide in patients who do not receive oral P2Y12 due to its rapid effect during the transition to these drugs [33]. However, it can also be used for perioperative P2Y12 bridging [34]. These data are not available in our study because there is no approval in Türkiye. Switching patients using Cangrelor to oral P2Y12 inhibitors can be done immediately after cangrelor discontinuation, as in our study. However, clopidogrel can be started 30 min before discontinuing cangrelor [35].

This study reported low incidence rates of bleeding within 48 h. In particular, the number of bleeding events in patients treated with BARC was ≥3 (1.7%) even though more than half of these patients received glycoprotein IIb/IIIa inhibitors. The bleeding rates were low at 1 month and were comparable between STEMI and other groups. These results are consistent with those of the multicenter ARCANGELO study in Italy, which reported similar bleeding rates in 324 patients [36,37]. These findings suggest the need for avoiding glycoprotein IIb/IIIa inhibitors in cangrelor-treated patients and implementing gastrointestinal protective strategies, especially in those with a history of gastrointestinal bleeding. Subgroup analyses from the CHAMPION trials reported similar ischemic outcomes but reported increased bleeding risk with glycoprotein IIb/IIIa inhibitors [38,39].

The ARCANGELO study, which investigated cangrelor in P2Y12-naïve ACS patients, reported that the 1-month MACE rate was 1.4%. The majority of events occurred in the first 48 h, with a 1.0% MACE rate in the first 48 h. Cangrelor was initiated for emergency reasons in 89% of the study population, and the rates of patients with cardiogenic shock and cardiac arrest were quite low [36,37]. In our study, 40% required inotropes, and approximately one-third experienced in-hospital mortality. Bleeding rates in our study, which was higher risk than the patients in the ARCANGELO study, were similar to this study. These results indicate that cangrelor is safe in high-risk patients.

Clopidogrel was used as a P2Y12 inhibitor in more than half of the patients in this study, which is contrary to guideline recommendations. The TURKMI study in Türkiye also reported clopidogrel as the most commonly used P2Y12 inhibitor in ACS. This may be due to the need for additional conditions for the use of prasugrel and ticagrelor in Türkiye (approved only in troponin-positive ACS patients), physician preference, or the high risk of bleeding in these patients [28]. The bleeding or ischemic events were not significantly different between clopidogrel-treated and ticagrelor/prasugrel-treated groups in the early period (48 h–1 month). However, clopidogrel was associated with increased bleeding tendency, while ticagrelor/prasugrel was associated with increased ischemic event tendency. These findings must be confirmed in studies with a large sample size.

The bleeding rates were similar in patients aged ≥75 years and those with CKD, which was consistent with the findings of previous studies examining the safety of cangrelor in these groups [40]. Age and potent P2Y12 inhibitor use were significant bleeding predictors, emphasizing the need for gastrointestinal protection, avoidance of glycoprotein IIb/IIIa inhibitors, heparin dose adjustment, and radial access in elderly patients and those on potent P2Y12 inhibitors [41,42]. Hypertension was not a bleeding predictor, which may be due to confounding factors, such as medication choice or clinical management [43].

Given the limited number of bleeding events (n = 26), the multivariate logistic regression model included seven predictors (age, hypertension, diabetes mellitus, CKD, Killip class, P2Y12 inhibitor type, and sex). This event-to-variable ratio is below the conventional threshold for robust logistic regression, and therefore, the results should be interpreted with caution. The model findings should be viewed as exploratory and hypothesis-generating rather than confirmatory.

In this study, the femoral approach was used in the majority of cases. While current literature recommends the radial approach, the femoral approach is still frequently preferred in our country due to its ease of access and time savings, especially in patients with cardiogenic shock [44]. Although, in the CHAMPION PHOENIX cohort, bleeding rates were lower in the radial intervention group, our study shows that cangrelor is safe to use in patients undergoing the femoral approach [45]. Approximately half of the patients had two or more lesions, and 14% developed contrast-induced nephropathy, suggesting the complexity of PCI procedures in this cohort. These findings are consistent with those of recent studies examining the efficacy and safety of cangrelor in complex PCI [46,47,48,49]. Allergic reactions or adverse events requiring cangrelor discontinuation were not observed, further supporting its safety [36,50]. The majority of our study population consists of patients with cardiogenic shock and cardiac arrest. In these patients, oral intake and gastrointestinal absorption are impaired, and the risk of bleeding also increases, and intravenous ASA and cangrelor with parenteral antiplatelet strategy should be considered as the best way to provide timely DAPT [49].

The multicenter, real-world design of this study is a significant strength. However, the retrospective nature of this study and the restriction of cangrelor use to high-risk patients due to reimbursement policies limit the generalizability of results to the broad CAD population. Large-scale studies must be performed with all CAD groups to validate these findings. The lack of standardized angiographic data such as clot burden and lesion scoring is also a limitation. Therewithal, the retrospective design reduces the level of evidence of the study data because there is no randomized comparison of cangrelor with any other drug.

## 6. Conclusions

This is the first multicenter, large-cohort study on cangrelor use in Türkiye. The findings of this study indicate the efficacy and safety of cangrelor use in high-risk patients with complex clinical and lesion characteristics. Although the bleeding rates were low, glycoprotein IIb/IIIa inhibitors should be avoided, and gastrointestinal protective strategies must be implemented. Cangrelor was safe in the following patient groups: age ≥ 75 years, CKD, and undergoing femoral access. These results suggest that cangrelor use should be expanded in countries with health policy restrictions, such as Türkiye.

## Figures and Tables

**Table 1:** Demographic and clinical characteristics of the study participants.

Characteristic	Evaluable Patients (n = 411)
Age (years)	63.8 ± 12.7
Sex	
Female	98 (24%)
Male	313 (76%)
Body mass index (kg/m^2^)	28.3 ± 4.4
Type of CAD	
STEMI	268 (65%)
NSTEMI	100 (24%)
UA	10 (2.4%)
CCS	33 (8.0%)
Indication for cangrelor	
Cardiogenic shock	141 (34%)
Vomiting	70 (17%)
Intubation	113 (28%)
Dysphagia	19 (4.6%)
Complex coronary intervention	62 (15%)
Smoking	193 (53%)
Hypertension	255 (62%)
Diabetes mellitus	189 (46%)
Hyperlipidemia	200 (49%)
Family history	163 (46%)
Prior MI	106 (26%)
Prior PCI	111 (27%)
Peripheral artery disease	20 (4.9%)
Atrial fibrillation	35 (8.5%)
Cerebrovascular disease	27 (6.7%)
COPD	38 (9.2%)
Gastrointestinal disease	5 (1.3%)
CKD	76 (18%)
Heart rate (bpm)	86.0 [70.0; 105.0]
Systolic BP (mmHg)	100.0 [80.0; 125.0]
Diastolic (mmHg)	60.0 [45.0; 74.0]
Killip class at admission	
1	185 (50%)
2	52 (14%)
3	22 (5.9%)
4	111 (30%)
Hemoglobin (g/dL)	13.0 ± 2.2
eGFR (mL/min/1.73 m^2^)	71.0 [50.0; 89.0]
LVEF (%)	40.0 [30.0; 52.5]

Abbreviations: STEMI: ST-elevation myocardial infarction; CCS: chronic coronary syndrome; COPD: chronic obstructive pulmonary disease; CKD: chronic kidney disease; eGFR: estimated glomerular filtration rate; LVEF: left ventricular ejection fraction; MI: myocardial infarction; NSTEMI: non-ST-elevation myocardial infarction; PCI: percutaneous coronary intervention; UA: unstable angina. Data summarized as mean ± standard deviation, median [25th percentile; 75th percentile], n (%).

**Table 2:** Detailed characteristics of percutaneous coronary intervention.

Characteristic	Evaluable Patients (n = 411)
Catheter access site	
Femoral	298 (72%)
Radial	114 (28%)
No. of diseased vessels	
1	172 (41%)
2	139 (34%)
3	95 (23%)
4	4 (1.0%)
5	1 (0.2%)
Culprit lesion vessel	
LAD	216 (57%)
CX	66 (17%)
RCA	94 (25%)
LMCA	3 (0.8%)
Diagonal	3 (0.8%)
Bifurcation lesion	71 (17%)
Type of the implanted stent	
DES	376 (95%)
BMS	11 (2.8%)
DCB	7 (1.8%)
Stent brand/model	
Everolimus	186 (54%)
Sirolimus	73 (21%)
Rapamisin	50 (14%)
Biolimus	13 (3.8%)
Zotarolimus	10 (2.9%)
Stent diameter (mm)	3.0 [2.8; 3.5]
Total stent length (mm)	29.0 [20.0; 44.0]
No. of implantend stents	1.0 [1.0; 2.0]
PCI duration (min)	39.0 [26.0; 55.0]
Contrast volume (mL)	150.0 [110.0; 220.0]
Infusion time (min)	2.0 [2.0; 2.0]
P2Y12 inhibitor type	
Clopidogrel	231 (57%)
Ticagrelor	172 (42%)
Prasugrel	4 (1.0%)
P2Y12 initiation time (min)	1.5 [1.0; 2.0]
GP IIb/IIIa use	44 (11%)

Abbreviations: PCI: Percutaneous coronary intervention; BMS: bare-metal stent, CX: circumflex artery, DCB: drug-coated balloon, DES: drug-eluting stent, LAD: left anterior descending artery, LMCA: left main coronary artery, RCA: right coronary artery. Data summarized as median [25th percentile; 75th percentile], n (%).

**Table 3:** Detailed description of the observed bleeding events.

Bleeding Event	Evaluable Patients (n = 411)
Bleeding severity at 48 h (BARC)	
No bleeding	383 (94.0%)
Type 1	16 (3.9%)
Type 2	3 (0.7%)
Type 3a	6 (1.5%)
Type 5a	1 (0.2%)
Bleeding severity at 48 h (GUSTO)	
No bleeding	386 (94.0%)
Mild	15 (3.6%)
Moderate	7 (1.7%)
Severe	3 (0.7%)
Bleeding site	
Vascular access site	8 (2.0%)
Hematuria	7 (1.7%)
Oral cavity	4 (1.0%)
Gastro-intestinal system	3 (0.7%)
Pericardium	1 (0.2%)
Lung	1 (0.2%)
Intracranial	1 (0.2%)
Aortic dissection	1 (0.2%)
Transfusion required	15 (3.7%)
Surgical intervention required	1 (0.2%)
Bleeding severity at 1 month (BARC)	
No bleeding	273 (97.0%)
Type 1	6 (2.1%)
Type 2	1 (0.4%)
Type 3a	1 (0.4%)
Type 5a	0 (0.0%)
Bleeding severity at 1 month (GUSTO)	
No bleeding	273 (97.0%)
Mild	6 (2.1%)
Moderate	1 (0.4%)
Severe	1 (0.4%)
Any bleeding during follow-up	2 (0.8%)

Abbreviations: BARC: Bleeding Academic Research Consortium, GUSTO: Global Use of Strategies to Open Occluded Coronary Arteries. Data summarized as n (%).

**Table 4:** Distribution of Bleeding Events According to BARC and GUSTO Classifications at 48 h and 1 Month, Stratified by ACS Subtype.

Timepoint	Scale	Category	NSTEMI	STEMI	UA
48 h	BARC	Type 1	5 (71.4%)	10 (55.6%)	1 (50%)
		Type 2	1 (14.3%)	2 (11.1%)	1 (50%)
		Type 3a	1 (14.3%)	5 (27.8%)	–
		Type 5a	–	1 (5.6%)	–
48 h	GUSTO	Mild	5 (83.3%)	8 (47.1%)	–
		Moderate	1 (16.7%)	6 (35.3%)	2 (100%)
		Severe	–	3 (17.6%)	–
1 month	BARC	Type 1	3 (75%)	3 (75%)	–
		Type 2	–	1 (25%)	–
		Type 3a	1 (25%)	–	–
1 month	GUSTO (corrected)	Mild	2 (66.7%)	1 (33.3%)	2 (100%)
		Moderate	1 (33.3%)	2 (66.7%)	–
		Severe	–	–	–

a. Percentages represent the proportion of bleeding events within each ACS subtype (NSTEMI, STEMI, UA) for the corresponding timepoint and bleeding category. b. BARC bleeding definitions: Type 1 = minor bleeding not requiring clinical evaluation; Type 2 = overt bleeding requiring medical attention; Type 3a = overt bleeding with hemoglobin decrease of 3–5 g/dL; Type 5a = probable fatal bleeding. c. GUSTO bleeding definitions: Mild = minimal bleeding; Moderate = bleeding requiring transfusion but without hemodynamic compromise; Severe = life-threatening bleeding associated with significant hemodynamic compromise. d. “–” indicates that no bleeding events were observed in that category for the respective ACS subtype.

**Table 5:** Determinants of 48 h bleeding.

Variables	OR (95% CI)	*p*
Age (for every 1 year)	1.04 (1.01–1.08)	0.018
Hypertension	0.32 (0.14–0.73)	0.007
Potent P2Y12 (Ticagrelor/Prasugrel)	2.17 (0.96–4.92)	0.064
Cardiogenic shock (Killip 4)	1.55 (0.65–3.71)	0.326
Diabetes Mellitus	1.47 (0.64–3.39)	0.361
CKD	1.09 (0.39–3.05)	0.876
Gender (female)	0.93 (0.35–2.44)	0.878

Abbreviation: CI: Confidence interval, CKD: Chronic kidney disease; OR: Odds ratio.

## Data Availability

The datasets analyzed or generated during the current study are available from the corresponding author on reasonable request. S.A has full access to all the data in the study and takes responsibility for the integrity of the data and accuracy of the data analysis.
